# Supplementary material for: An antiferromagnetic spin phase change memory
Source: Nat Commun. 2024 Jun 11;15:4978. doi: 10.1038/s41467-024-49451-2 (PMC11166633; doi:10.1038/s41467-024-49451-2)
Supplement: Supplementary file 1 — Supplementary Information [file 41467_2024_49451_MOESM1_ESM.docx]

Supplementary Information

An Antiferromagnetic Spin Phase Change Memory

*Han Yan^1,^*^#^*, Hongye Mao^1,^*^#^*, Peixin Qin^1,^*, Jinhua Wang^2^, Haidong Liang^3^, Xiaorong Zhou^1^, Xiaoning Wang^1^, Hongyu Chen^1^, Ziang Meng^1^, Li Liu^1^, Guojian Zhao^1^, Zhiyuan Duan^1^, Zengwei Zhu^2^, Bin Fang^4^, Zhongming Zeng^4^, Andrew A. Bettiol^3^, Qinghua Zhang^5,^*, Peizhe Tang^1,6,^*, Chengbao Jiang^1,^*, Zhiqi Liu^1,^**

^1^School of Materials Science and Engineering, Beihang University; Beijing 100191, China

^2^Wuhan National High Magnetic Field Center, Huazhong University of Science and Technology; Wuhan 430074, China

^3^Centre for Ion Beam Applications (CIBA), Department of Physics, National University of Singapore; Singapore 117542, Singapore

^4^Key Laboratory of Multifunctional Nanomaterials and Smart Systems, Suzhou Institute of Nano-Tech and Nano-Bionics, Chinese Academy of Sciences, Suzhou 215123, China

^5^Beijing National Laboratory for Condensed Matter Physics, Institute of Physics, Chinese Academy of Sciences; Beijing 100190, China

^6^Max Planck Institute for the Structure and Dynamics of Matter, Center for Free Electron Laser Science; Hamburg 22761, Germany

*Corresponding author.

Email: [qinpeixin@buaa.edu.cn](mailto:qinpeixin@buaa.edu.cn); [zqh@iphy.ac.cn;](mailto:zqh@iphy.ac.cn;) [peizhet@buaa.edu.cn](mailto:peizhet@buaa.edu.cn); [jiangcb@buaa.edu.cn](mailto:jiangcb@buaa.edu.cn); [zhiqi@buaa.edu.cn](mailto:zhiqi@buaa.edu.cn)

^#^These authors contributed equally to this work.

**This PDF file includes:**

Supplementary Figures 1 to 7

Supplementary Notes 1 to 2

**Supplementary Figures**

**Supplementary Figure 1**

**
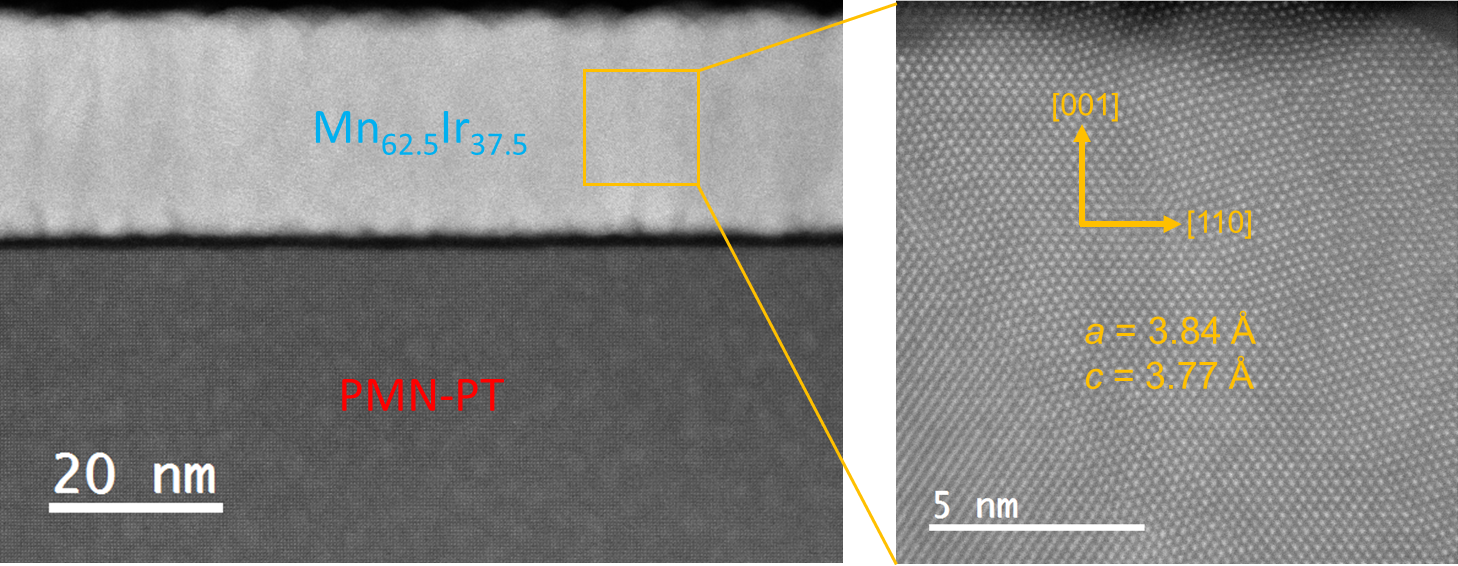
**

**Supplementary Fig. 1 |** Cross-section transmission electron microscopy image of a virgin state 26-nm-thick Mn-Ir/PMN-PT heterostructure.

**Supplementary Figure 2**


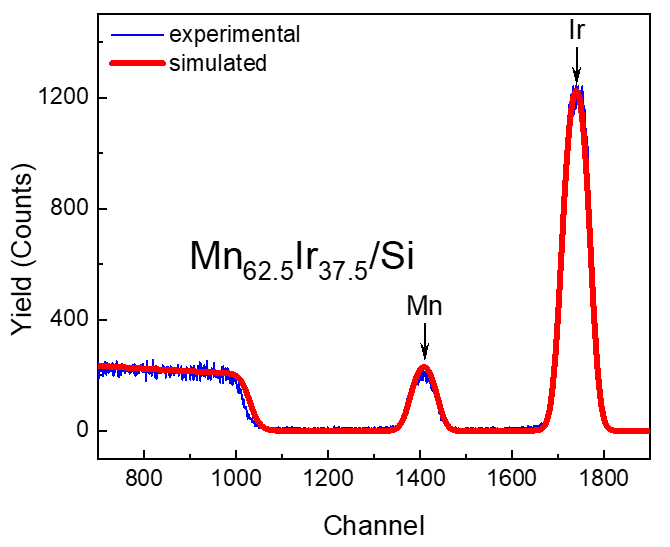


**Supplementary Fig. 2 |** Rutherford backscattering and fitting curves of the Mn-Ir/Si heterostructure.

**Supplementary Figure 3**


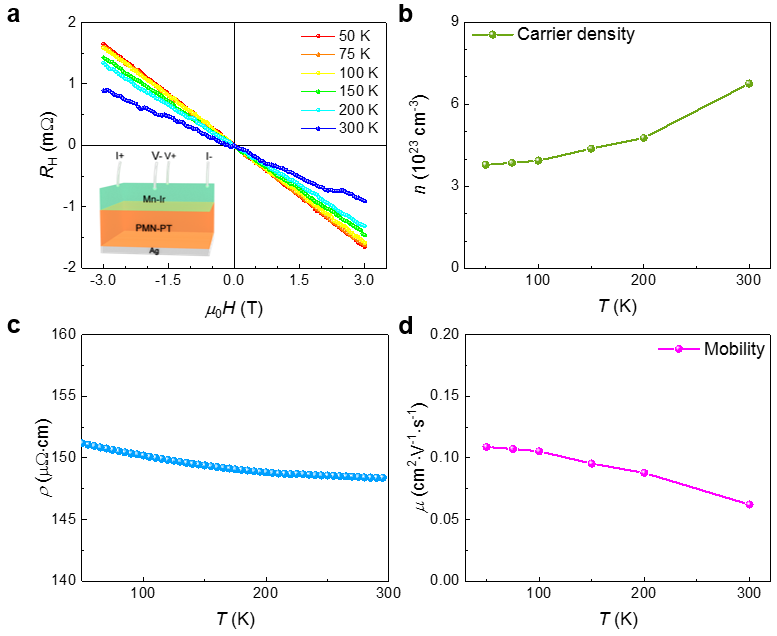


**Supplementary Fig. 3 |** **Transport properties of the as-grown Mn-Ir/PMN-PT heterostructure.** **a** Hall resistance of the Mn-Ir/PMN-PT heterostructure of the virgin state from 50 to 300 K. Inset: Schematic of the Hall measurement geometry. **b-d** Temperature-dependent carrier density, resistivity and carrier mobility of the as-grown Mn-Ir/PMN-PT heterostructure from 50 to 300 K, respectively.

**Supplementary Figure 4**

**
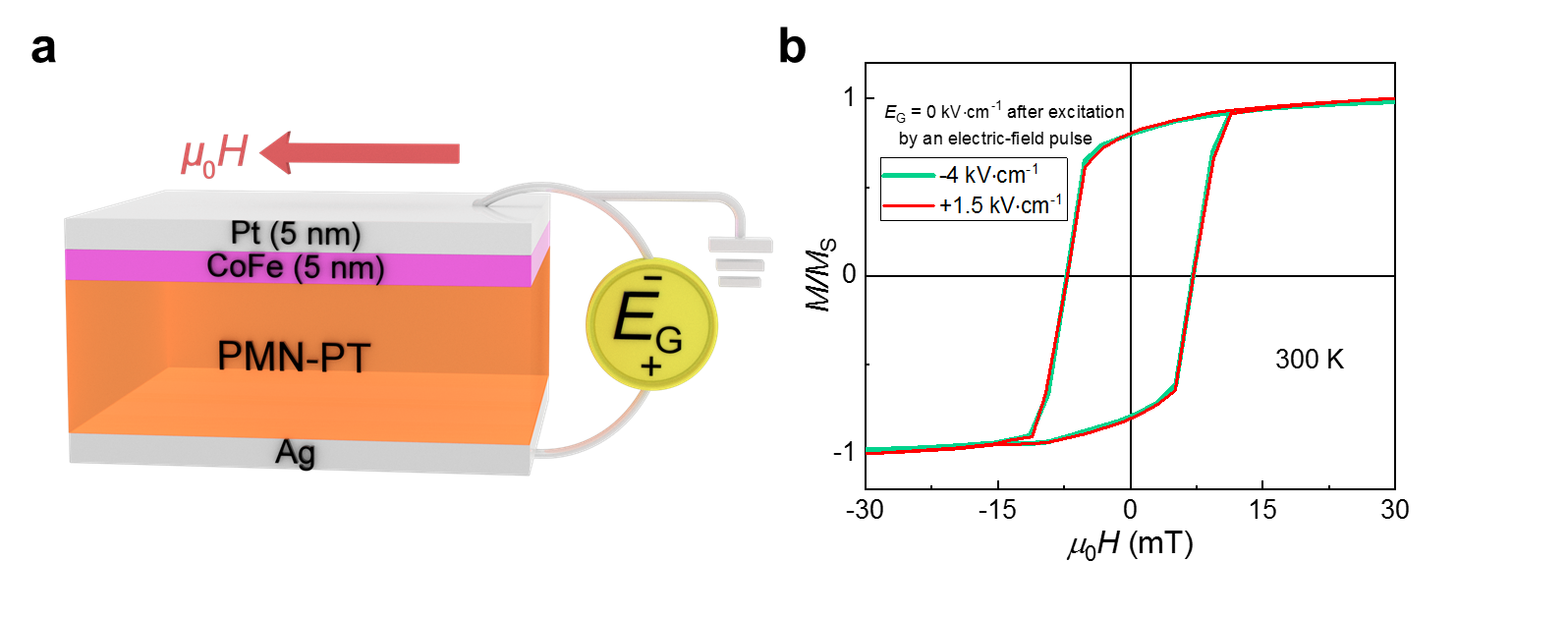
**

**Supplementary Fig. 4 |** **Magnetic measurements in a Pt/CoFe/ PMN-PT heterostructure after applying positive/negative electric-field pulses.** **a** Illustrations of a Pt/CoFe/PMN-PT heterostructure, the magnetization measurement geometry and the gate-electric-field-switching geometry. **b** Room-temperature magnetic hysteresis loop of the heterostructure shown in (**a**) after applying positive/negative electric-field pulses.

**Supplementary Figure 5**


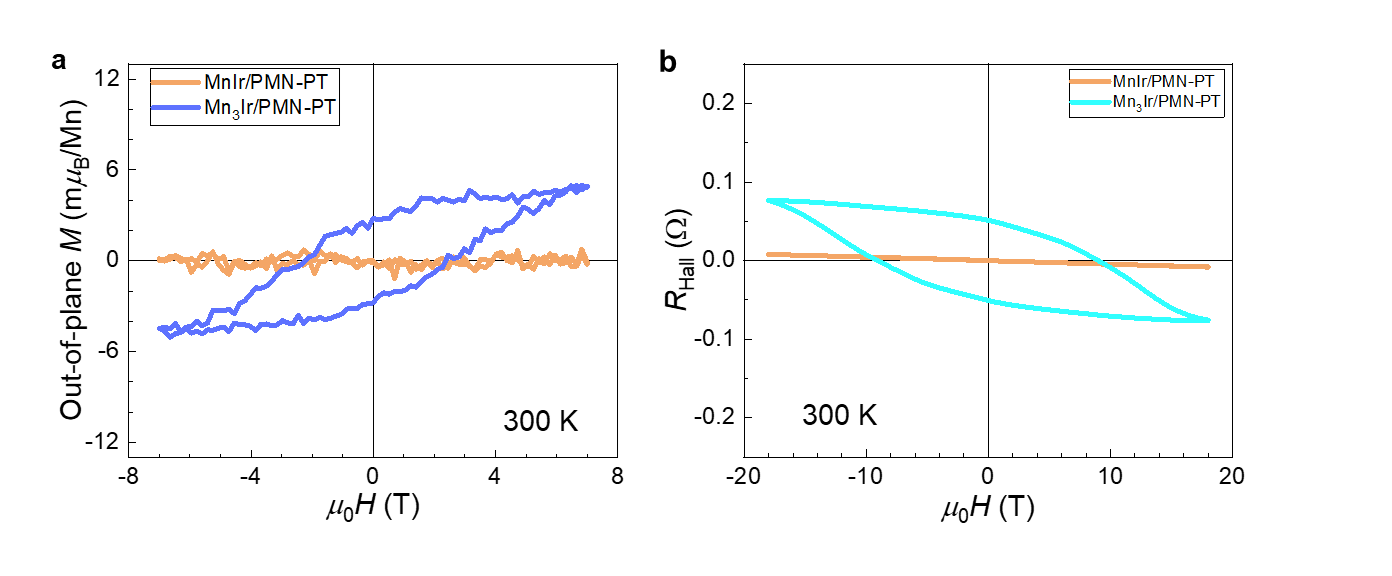


**Supplementary Fig. 5 |** **Magnetic and Hall measurements in the *L*1_0_ MnIr/PMN-PT and *L*1_2_ Mn_3_Ir/PMN-PT heterostructures.** **a, b** Room-temperature out-of-plane magnetization (**a**) and Hall resistance (**b**) of the *L*1_0_ MnIr/PMN-PT and *L*1_2_ Mn_3_Ir/PMN-PT heterostructures.

**Supplementary Figure 6**


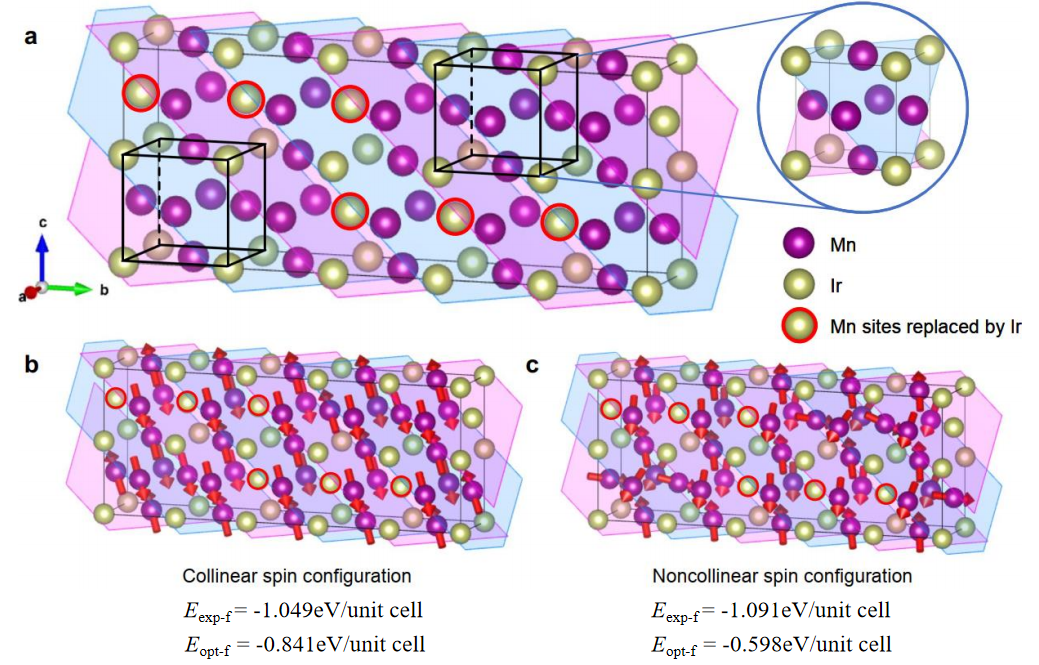


**Supplementary Fig. 6 |** **Theoretical modeling of the possible atomic and spin configurations. a** A relaxed crystal structure pertaining to a composition-boundary Mn-Ir film. **b** The collinear antiferromagnetic phase of the experimental lattice parameters and the fully optimized lattice parameters in (**a**) with the formation energy (*E*_exp-f_, *E*_opt-f_). **c** The noncollinear antiferromagnetic phase of the experimental lattice parameters and the fully optimized lattice parameters in (**a**) with the formation energy (*E*_exp-f_, *E*_opt-f_).

**Supplementary Figure 7**


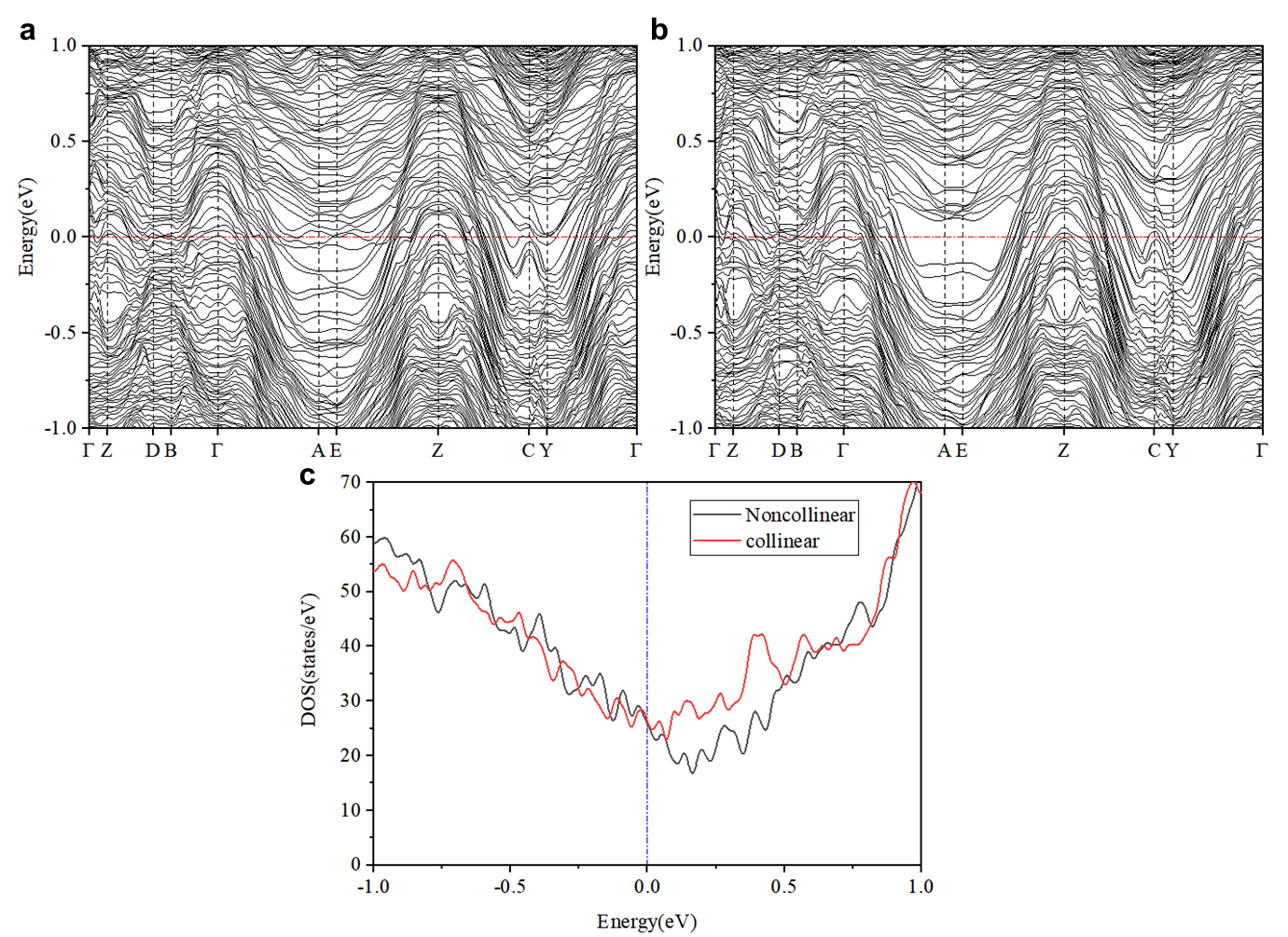


**Supplementary Fig. 7 |** **The band structures and density of states for both collinear and noncollinear phases. a** The band structure for the collinear phase. **b** The band structure for the noncollinear phase. **c** The comparison of the density of states between collinear and noncollinear phases.

**Supplementary Notes**

**Supplementary Note 1. Theoretical calculations**

The calculations of formation energy were performed by using the Vienna *ab*-*initio* Simulation Package (VASP)^1^ with the projected augmented plane-wave (PAW) method^2^. The exchange-correlation functionals were applied as Perdew-Burke-Ernzerhof scheme^3^ and the plane-wave energy cutoff was set to 500 eV.

In order to build the theoretical model of Mn_1-_*_x_*Ir*_x_* (*x* = 35%-40%) intermetallic, the supercells were constructed based on the c-axis compressed Mn_3_Ir lattice and part of the Mn atoms were replaced by Ir atoms in order to satisfy the atom ratio. To balance the calculation cost and precision, the supercell was chosen as 1 × 5 × 2, and the lattice parameters are *a* = 3.84 Å, *b* = 3.84 × 5 = 19.2 Å, *c* = 3.77 × 2 = 7.54 Å, based on the experimental data. Since the experimental result reported the lattice parameters were equivalent in the $a$ and $b$ directions, it is reasonable that the supercell fixed the freedom of the $a$ direction and only extend in the *b* and *c* directions to fully relax the atoms.

Besides, to satisfy the requirement of atom ratio, 6 Mn atoms are replaced into Ir atoms so that the atom ratio of supercells can be 24:16, *i.e.*, *x* = 40 %. However, because of the large number of structure candidates, it is impossible to directly identify which alternative is the most stable structure by DFT calculations. Taking the atom relaxation degree of freedom and the atom radius into consideration, it is possible that *β* sites (Supplementary Note Fig. 1) would take precedence over $\alpha$ sites when being replaced. Considering the transformation of the Hall effect in the experimental data, during the experiment, it is plausible that there are quasi-Mn_3_Ir nano-structures in the supercells, and the transformation of the Hall effect may derive from the spin phase change of the quasi-Mn_3_Ir nano-structure.

Based on the concepts discussed above, the number of supercell alternatives is narrowed down, and consequently we are able to propose a possible supercell structure as illustrated in Supplementary Fig. 6a. The simulation was performed with 12 × 2 × 6 Monkhorst-Pack grid, and the collinear magnetic moments during relaxation were set based on the pure MnIr and Mn_3_Ir antiferromagnetic phases. Remarkably, the quasi-Mn_3_Ir nano-structure is transformed from tetragonal into quasi-cubic, which supports the anomalous Hall effect typically observed in the *L*1_2_-type noncollinear antiferromagnetic Mn_3_Ir.

Subsequently, SOC calculations were performed on the relaxed supercell to analyze the formation energy variation caused by the difference of the spin configuration, especially between the collinear spin and the noncollinear spin configurations. The collinear spin configuration is the same with the spin configuration used in relaxation, and the direction was inclined away from the *c*-axis, as shown in Supplementary Fig. 6b. As for the noncollinear spin configuration, several configurations (based on pure MnIr and Mn_3_Ir antiferromagnets) were tested and finally a spin configuration shown in Supplementary Fig. 6c was identified. The formation energy (or formation enthalpy) per unit cell (1/10 of supercell) was calculated according to the following formula:

$$E_{f}=\frac{E_{tot}-n_{Mn}E_{Mn}-n_{Ir}E_{Ir}}{10}$$

There were 24 Mn atoms and 16 Ir atoms in a supercell, which means *n*_Mn_ = 24 and *n*_Ir_ = 16. *E*_Mn_ and *E*_Ir_ are the average total energy in DFT calculations for each Mn and Ir atom, respectively.

Interestingly, the difference of the formation energy between the collinear and noncollinear spin configurations is so small (~42 meV/unit cell) that a subtle energy excitation provided by small piezoelectric strain can lead to the reversible interconversion of the two spin configurations, which explained the possible physical origin of the resistance/anomalous Hall resistance modulation by piezoelectric strain.

Regarding the DFT calculation details, the tests of DFT parameters were performed on Mn_3_Ir and MnIr intermetallic, and parameters such as *k*-points and energy cutoff were consistent during all the calculations. Based on calculations of Mn_3_Ir unit cell, 12 × 12 × 12 *k*-point grid and 500 eV energy cutoff were enough to converge the formation energy. In the following calculations of supercells, the density of *k*-points was kept similar and the energy cutoff was kept unchanged (500 eV).

Besides, assuming there was a *c*-axis compressed Mn_3_Ir unit cell, the Mn sites would be separated into two different types. For easier description, the sites could be defined as *α*, *β* and *γ* sites, as shown in Supplementary Note Fig. 1, and the *β* and *γ* sites were equivalent due to the equal *a* and *b* lattice constants.


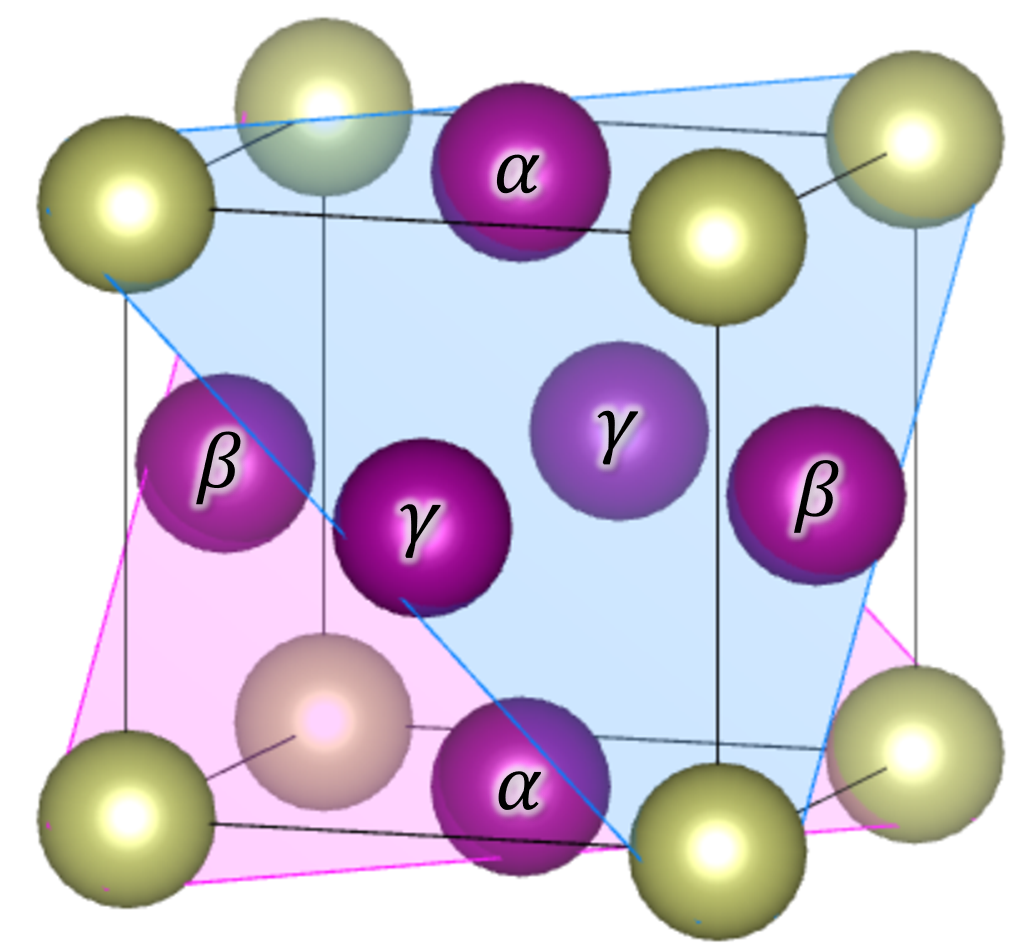


**Supplementary Note Fig. 1** | Schematic of Mn atoms at three equivalent sites for a Mn_3_Ir unit cell compressed along the out-of-plane *c* direction.

To reflect the effects of piezoelectric strain, noncollinear SOC calculations were performed on a *c*-axis compressed Mn_3_Ir unit cell, and resulted in a different spin configuration from that of the *L*1_2_-type Mn_3_Ir, as shown in Supplementary Note Fig. 2. Our calculations found that it is less likely to achieve a collinear spin configuration from the self-consistent calculations of *c*-axis compressed pure Mn_3_Ir, which suggests the absence of the segregation of Mn_3_Ir in a composition-boundary Mn_1-_*_x_*Ir*_x_* (*x* = 35%-40%) intermetallic film. However, quasi-Mn_3_Ir and quasi-MnIr nano-structures could coexist in the supercell (Supplementary Fig. 6a), which would facilitate the reversible interconversion of spin configurations.


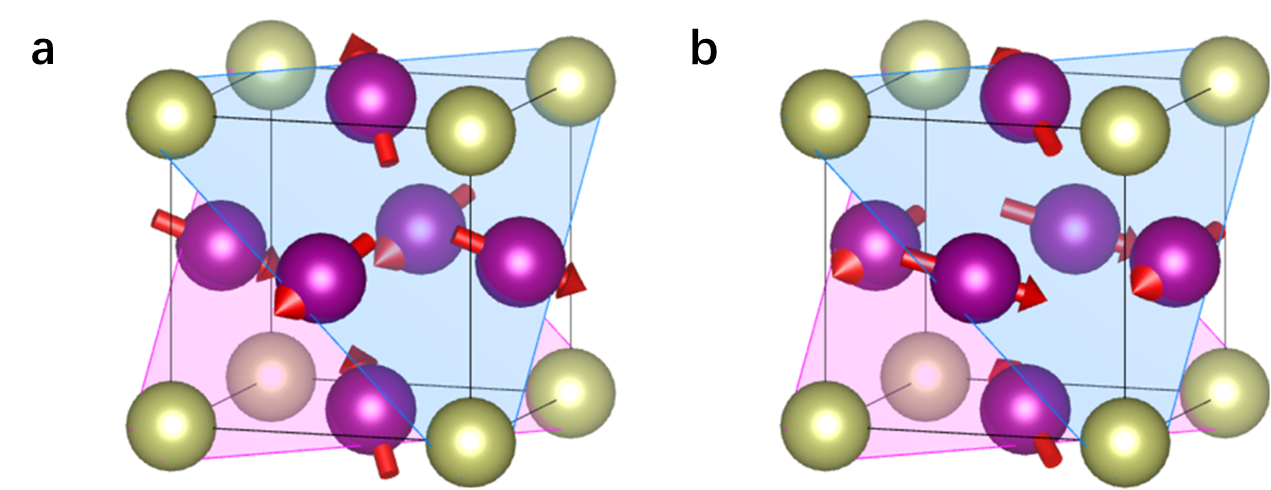


**Supplementary Note Fig. 2** | **Spin structures of different Mn_3_Ir cells. a,** Schematic of the spin structure for a cubic Mn_3_Ir cell. **b,** Schematic of the spin structure for a tetragonal Mn_3_Ir cell compressed along the *c*-axis.

On the other hand, we optimized the lattice structure of Mn_3_Ir by using the GGA-PBE exchange-correlation functional in the framework of DFT calculations. It is found that the optimized lattice constant (*a*_opt_ = 3.69Å) is much smaller than the experimental value (*a*_exp_ = 3.84 Å). Then, we used the optimized lattice to construct the supercell for Ir-Mn system, in which some Mn atoms were substituted by the Ir atoms. The optimized lattice parameters for the supercell are *a* = 3.688 Å, *b* = 18.44 Å, and *c* = 7.376 Å, in contrast to the experimental lattice parameters of *a* = 3.84 Å, *b* = 19.2 Å, and *c* = 7.54 Å. Then we compared the formation energy for these structures with collinear and noncollinear magnetic configurations. The calculated results are shown in Supplementary Fig. 6b, c. Within the experimental lattice parameters, the noncollinear phase exhibits lower energy, while within a fully optimized lattice parameters, the collinear phase exhibits lower energy. Such results clearly demonstrate that the formation energy of Mn-Ir system can be strongly remodulated by the strain, leading to different spin configurations.

Considering all the above aspects, the antiferromagnetic spin phase change scenario controlled by an external small piezoelectric strain is physically feasible for a composition-boundary Mn-Ir film. Accordingly, such a spin phase change leads to giant nonvolatile longitudinal and transverse resistance modulations and thus enables the excellent memory device applications.

**Supplementary Note 2. Ferroelectric substrates**

0.7PbMg_1/3_Nb_2/3_O_3_-0.3PbTiO_3_ (PMN-PT) is a type of ferroelectric oxide formed by the solid solution of two perovskite ferroelectrics, namely the relaxor ferroelectric PbMg_1/3_Nb_2/3_O_3_ (PMN) and the ordinary ferroelectric PbTiO_3_ (PT). PMN-PT possesses a pseudo-cubic perovskite structure with lattice constants of *a* = *b* = *c* = 4.02Å^4^. Furthermore, PMN-PT single crystals exhibit excellent ferroelectric, piezoelectric and dielectric properties. For instance, it demonstrates a piezoelectric constant *d*_33_ up to 2500 pC/N, remanent polarization strength *P*_r_ larger than 50 µC/cm^2^, and dielectric constant up to 38000 (ref. 5).

When in an unpolarized state, the electric dipoles within the PMN-PT single crystal are randomly oriented. With an increasing electric field applied to the crystal, these initially disordered dipoles tend to align along the direction of the field, inducing a contraction of the crystal lattice perpendicular to the electric field and an expansion parallel to the electric field. Upon application of a bipolar electric field exceeding the coercive field strength, the strain within the PMN-PT single crystal exhibits a characteristic butterfly-shaped response^6^.

On the other hand, piezoelectric strain in PMN-PT ferroelectrics originates from non-180° ferroelastic switching, there are multiple switching paths (180°/109°/71°) for ferroelectric polarization upon reversing the electric fields. That is, there could be inequivalent switching possibilities for different switching paths for positive and negative electric fields. Hence, there is a residual strain after the electric field is removed, manifesting as an asymmetric butterfly-shape strain curve, which can be used to achieve non-volatile modulation^7,8^. Moreover, applying an electric field of one polarity smaller than its coercivity field, so that the switching on one polarity is not completed, can also create asymmetric strains, and generate non-volatile strain states at zero electric fields^9^.

Commonly, (001)-, (011)- and (111)-oriented PMN-PT single crystal are employed as ferroelectric substrates. In this work, we utilized (001)-oriented PMN-PT single-crystal substrates with an in-plane size of 2.5 × 5 mm^2^ and an out-of-plane thickness of 0.3 mm. Before the deposition of the Mn-Ir thin films, the PMN-PT substrates did not undergo any electric-field excitation.

**Supplementary References**

1. Kresse, G. & Furthmüller, J. Efficient iterative schemes for *ab initio* total-energy calculations using a plane-wave basis set. *Phys. Rev. B* **54**, 11169 (1996).
2. Blöchl, P. E. Projector augmented-wave method. *Phys. Rev. B* **50**, 17953 (1994).
3. Perdew, J. P. Burke, K. & Ernzerhof, M. Generalized gradient approximation made simple. *Phys. Rev. Lett.* **77**, 3865 (1996).
4. Noheda, B. Cox, D. E. Shirane, G. Gao, J. & Ye, Z.-G. Phase diagram of the ferroelectric relaxor (1-*x*)PbMg_1/3_Nb_2/3_O_3_-*x*PbTiO_3_. *Phys. Rev. B* **66**, 054104 (2002).
5. Fang, B. J. *et al.* Preparation of Pb(Mg_1/3_Nb_2/3_)O_3_-PbTiO_3_ ceramics by reaction-sintering method and their electrical properties. *Eur. Phys. J. Appl. Phys.* **57**, 30101 (2012).
6. Xu, M. *et al.* Progresses of magnetoelectric composite films based on PbMg_1/3_Nb_2/3_O_3_-PbTiO_3_ single-crystal substrates. *Acta Phys. Sin.* **67**, 157506 (2018).
7. Zhang, S. *et al.* Electric-field control of nonvolatile magnetization in Co_40_Fe_40_B_20_/Pb(Mg_1/3_Nb_2/3_)_0.7_Ti_0.3_O_3_ structure at room temperature. *Phys. Rev. Lett.* **108**, 137203 (2012).
8. Wu, S. Z. *et al.* Strain-mediated electric-field control of exchange bias in a Co_90_Fe_10_/BiFeO_3_/SrRuO_3_/PMN-PT heterostructure. *Sci. Rep.* **5**, 8905 (2015).
9. Lee, Y. *et al.* Large resistivity modulation in mixed-phase metallic systems. *Nat. Commun.* **6**, 5959 (2015).
